# Challenges and Advances in Managing Thrombocytopenic Cancer Patients

**DOI:** 10.3390/jcm10061169

**Published:** 2021-03-11

**Authors:** Avi Leader, Liron Hofstetter, Galia Spectre

**Affiliations:** 1 Institute of Hematology, Davidoff Cancer Center, Rabin Medical Center, Petah Tikva 4941492, Israel; lironho@clalit.org.il (L.H.); galiasp1@clalit.org.il (G.S.); 2 Sackler School of Medicine, Tel Aviv University, Tel Aviv 6997801, Israel; 3 Cardiovascular Research Institute Maastricht (CARIM), Maastricht University, 6229 ER Maastricht, The Netherlands

**Keywords:** anticoagulation, antifibrinolytic, antiplatelet, cancer, thrombocytopenia, thrombopoietin receptor agonist, tranexamic acid

## Abstract

Cancer patients have varying incidence, depth and duration of thrombocytopenia. The mainstay of managing severe chemotherapy-induced thrombocytopenia (CIT) in cancer is the use of platelet transfusions. While prophylactic platelet transfusions reduce the bleeding rate, multiple unmet needs remain, such as high residual rates of bleeding, and anticancer treatment dose reductions/delays. Accordingly, the following promising results in other settings, antifibrinolytic drugs have been evaluated for prevention and treatment of bleeding in patients with hematological malignancies and solid tumors. In addition, Thrombopoeitin receptor agonists have been studied for two major implications in cancer: treatment of severe thrombocytopenia associated with myelodysplastic syndrome and acute myeloid leukemia; primary and secondary prevention of CIT in solid tumors in order to maintain dose density and intensity of anti-cancer treatment. Furthermore, thrombocytopenic cancer patients are often prescribed antithrombotic medication for indications arising prior or post cancer diagnosis. Balancing the bleeding and thrombotic risks in such patients represents a unique clinical challenge. This review focuses upon non-transfusion-based approaches to managing thrombocytopenia and the associated bleeding risk in cancer, and also addresses the management of antithrombotic therapy in thrombocytopenic cancer patients.

## 1. Introduction

**Figure 1:**
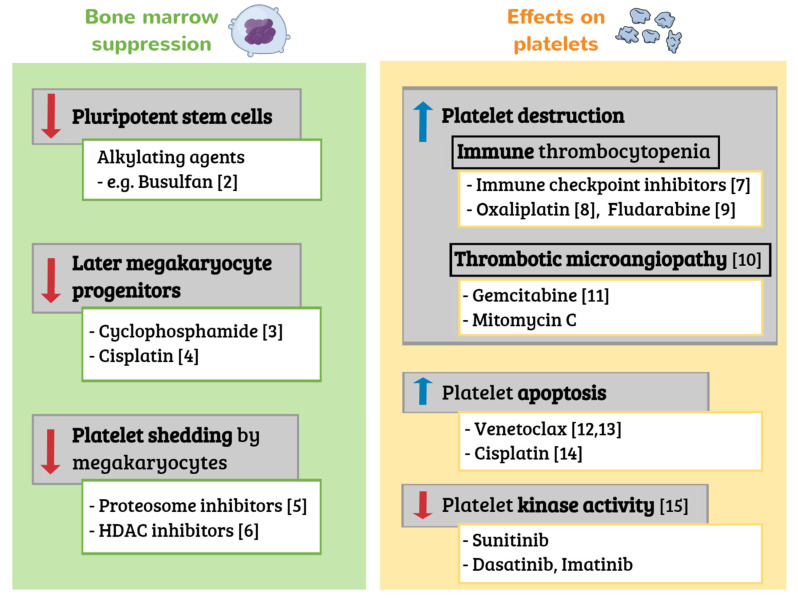
Selected mechanisms of drug induced thrombocytopenia in cancer. Examples of implicated drugs are given for each mechanism. *HDAC*, histone deacetylase.

Cancer patients have varying incidence, depth and duration of thrombocytopenia, depending on cancer type, anticancer treatment, bone marrow involvement and comorbidities [1]. For example, patients with hematological malignancies and those receiving carboplatin or oxaliplatin based chemotherapy regimens, have a higher risk of severe thrombocytopenia. Anticancer drugs can cause thrombocytopenia via various mechanisms [2,3,4,5,6,7,8,9,10,11,12,13,14,15], as previously reviewed [1] and as shown in Figure 1. While pancytopenia due to general bone marrow suppression is most common, some antineoplastic drugs, such as proteosome inhibitors used primarily in multiple myeloma, can cause isolated thrombocytopenia. Bortezomib, a first-generation proteasome inhibitor, was found to reduce the mean platelet number by approximately 60%, independent of the baseline platelet count [9]. Proteosome inhibitor associated thrombocytopenia has a cyclic, transient pattern [16,17]. The mechanism was first suggested to be related to the prevention of the activation of NF-κB which may potentially prevent platelet budding from megakaryocytes. Further studies found that the pharmacologic inhibition of proteasome activity blocks proplatelet formation, due to the upregulation and hyperactivation of the small GTPase RhoA, rather than NF-κB [18]. Although thrombocytopenia is commonly observed, there are only a few reports of serious bleeding complications with proteosome inhibitors [17,19].

Severe thrombocytopenia (<10 × 10^9^/L) is associated with an increased risk of bleeding in cancer [20,21]. However, individual platelet counts between 10 and 50 × 10^9^/L do not clearly predict bleeding [22,23,24]. Multiple other factors affect the bleeding risk, such as fever, sex, renal failure, liver dysfunction, hematocrit ≤25% and use of antithrombotic drugs [21,24,25]. These factors should be considered when assessing bleeding risk and addressed when modifiable. In addition, emerging data show that patients with cancer-associated thrombocytopenia have additional hemostatic defects, such as platelet and endothelial dysfunction, as well as coagulation abnormalities, such as hyperfibrinolysis [26,27,28].

The mainstay of managing severe chemotherapy-induced thrombocytopenia (CIT) in cancer is the use of platelet transfusions. In most cancer settings, platelet transfusions are indicated prophylactically when platelets counts are <10 × 10^9^/L or therapeutically when bleeding occurs with platelets below 50 × 10^9^/L [29]. Different platelet transfusion thresholds may be warranted in the context of invasive procedures, sepsis, autologous stem cell transplantation and chronic stable disease-related thrombocytopenia, among other scenarios [21,29]. While prophylactic platelet transfusions reduce the rate of WHO grade ≥2 bleeding [22,23], multiple unmet needs remain in patients with cancer and thrombocytopenia, including the following: high rates of bleeding despite platelet transfusion [22]; thrombocytopenia-driven anticancer treatment dose reductions or delay; platelet-transfusion refractoriness [30]; managing antithrombotic drugs when indicated. 

This review focuses upon non-transfusion-based approaches to managing thrombocytopenia and the associated bleeding risk in cancer, and also addresses the management of antithrombotic therapy in thrombocytopenic cancer patients. The topic of platelet transfusions in cancer patients has been previously reviewed [29] and is covered elsewhere in this issue of the Journal.

## 2. Managing Thrombocytopenia in Cancer

### 2.1. Antifibrinolytic Therapy

Tranexamic acid (TXA) and aminocaproic acid (EACA) are synthetic antifibrinolytic drugs that lead to the inhibition of the conversion of plasminogen to plasmin and to the decrease in the lysis of fibrin clots [31]. Antifibrinolytic therapy has been shown to aid in the management of bleeding in multiple clinical scenarios such as trauma, postpartum hemorrhage, menorrhagia, and surgical bleeding [32]. On the other hand, recent negative findings of a randomized controlled trial (RCT) of TXA in acute gastrointestinal bleeding and a higher rate venous thromboembolism (VTE) in the TXA arm, serve as a reminder that setting-specific evidence is needed [33]. In light of this, the utility of antifibrinolytic drugs in solid tumors and hematological malignancies has also been evaluated. This review focuses on CIT or cancer-related thrombocytopenia, outside the context of disseminated intravascular coagulation (DIC) [34,35].

#### 2.1.1. Solid Tumors

A number of small RCTs and retrospective cohort studies were performed to assess the effect of perioperative antifibrinolytics on bleeding during and after cancer surgery, in a variety of solid malignancies. The studies including patients with liver, prostate and gynecological cancer found a reduction in blood transfusion requirements during and after surgery in the TXA arms [36,37,38,39,40,41]. In contrast, antifibrinolytics did not influence bleeding outcomes in major orthopedic cancer surgery or in oncologic spinal canal, head and neck and neurosurgeries [42,43,44,45]. 

Data on the use of antifibrinolytics for the treatment of active bleeding in solid cancer is scarce and limited to case reports and series. Several case reports showed favorable bleeding outcomes with TXA in the management of bleeding from malignant mesothelioma with hemothorax [46], hemoptysis due to bronchogenic carcinoma [47] and DIC after a prostatic biopsy [48]. One small case series (n = 16) demonstrated high rates of bleeding control with TXA and EACA for cancer associated bleeding in the palliative care setting [49]. 

#### 2.1.2. Hematological Malignancies with Thrombocytopenia

EACA and TXA have been studied over the years in patients with hematological malignancies and thrombocytopenia (generally <50 × 10^9^/L) with or without bleeding. However, most of the studies are small, non-controlled and retrospective with various treatment protocols and doses. Since EACA and TXA have not been compared directly, the evidence on each of these drugs is presented separately, first as treatment and then as prophylaxis. 

##### Treatment of Bleeding

Two studies published in 1980 and 1985 evaluated the use of EACA for the control of bleeding in patients with various hematological malignancies and thrombocytopenia (<20 × 10^9^/L) and reported the improvement in bleeding control and a reduction in platelet transfusions [50,51]. An additional study published in 1998 evaluated 15 patients with bleeding and severe thrombocytopenia (platelets <20 × 10^9^/L) and showed a positive effect with a maximum EACA dose of 6 g/day [52]. In 2006, a retrospective study from the Cleveland clinic reviewed the use of EACA in 77 patients with thrombocytopenic (median platelet count = 7 × 10^9^/L) hemorrhage (mostly mucosal and gastrointestinal). The majority of patients had hematological malignancies, predominantly acute leukemia and non-Hodgkin lymphoma, and the remainder had solid tumors. The median average dose was also 6 g/day. Complete (i.e., cessation of bleeding at all sites) and partial response were achieved in 51 (66%) and 13 (17%) patients, respectively, resulting in a decrease in platelet and red blood cell transfusions [53]. In 2008, a retrospective study evaluating EACA in acute promyelocytic leukemia (APL) patients with coagulopathy defined as low alpha-2-antiplasmin levels suggested a lower incidence of severe hemorrhagic events [54].

A recent Dutch survey indicated that TXA is more commonly used for the control of bleeding in hematological malignancies than as prophylaxis [55], even though most studies of TXA were in the context of prophylaxis. There is currently scarce evidence supporting the use of this specific agent in this context. 

##### Prophylaxis of Bleeding

EACA as prophylactic treatment was evaluated in 1983 in a randomized controlled trial versus placebo in patients undergoing remission induction for acute leukemia. There was no difference in major bleeding between the two groups; however, there was a non-significant reduction in platelet transfusions in the EACA group [56]. A subsequent retrospective study in 2013 reported on EACA treatment in 44 chronically and severely thrombocytopenic patients with hematological malignancies and median platelet counts of 8 × 10^9^/L. EACA was associated with a low risk of major spontaneous bleeding and was well tolerated [57]. Two additional retrospective studies (2016, 2018) provided additional safety data by demonstrating no increase in VTE rates with EACA as prophylactic therapy in thrombocytopenic patients with hematological malignancy [58,59]. The PROBLEMA Trial, a phase II control trial study evaluating the effectiveness and safety of EACA versus prophylactic platelet transfusions to prevent bleeding in thrombocytopenic patients with hematological malignancies, is still ongoing [60]. Table 1 details the ongoing studies of antifibrinolytics in thrombocytopenic cancer patients.

Up until recently, only three small RCTs evaluating TXA in hematological malignancies had been published (1989 thru 1995) including patients with acute leukemia, APL, aplastic anemia and myelodysplastic syndrome (MDS) [61,62,63]. TXA was associated with fewer bleeding episodes and fewer transfusion requirements in two of these studies [61,62]. In the third pilot study evaluating eight patients with MDS and aplastic anemia, TXA did not appear to be efficacious [63]. It should be noted that only three patients completed the randomized portion of this study and that patients were used as their own control. In addition, a prospective single arm study published in 1990 demonstrated a significant reduction in platelet transfusion with prophylactic TXA during induction and consolidation treatment in acute leukemia, compared to historical controls [64]. Of concern is a case series of three allogenic hematopoietic stem cell transplant patients who developed veno-occlusive disease (VOD) shortly after receiving TXA. The authors postulated a role for plasminogen activator inhibitor-1 in the development of hepatic VOD and that TXA could trigger or accelerate this process [65]. 

Accordingly, a systematic review and meta-analysis of antifibrinolytics for the prevention of bleeding in patients with hematological disorders concluded that there is uncertainty whether antifibrinolytics reduce the risk of bleeding in such patients, due to the small number of participants and low quality of evidence [66]. The question whether or not antifibrinolytics increase the risk of thromboembolic events or other adverse events could not be answered. A subsequent meta-analysis published in 2017 evaluated the safety and efficacy of lysine analogues in a total of 1177 cancer patients (both hematological and solid tumors) [67]. No increased risk of venous thromboembolism was observed among patients receiving lysine analogues compared to controls, and their use significantly decreased blood loss and transfusion risk. 

The results of the randomized controlled A-TREAT trial, assessing prophylactic TXA administration in addition to routine platelet transfusion, were recently presented and published in abstract form [68]. The study included 165 patients in each arm and demonstrated that prophylactic TXA did not decrease the rate of WHO grade 2+ bleeding and did not change platelet and blood cell transfusions rates. Of note, the rate of central line occlusions was increased in the TXA arm. This preliminary publication suggests that TXA should not be currently used for preventing bleeding in addition to prophylactic platelet transfusions. The results of the sister TREAT-T trial conducted in the UK and Australia are eagerly anticipated (Table 1) [69]. Knowledge gaps not currently addressed by published or ongoing trials that we are aware of, include the use of antifibrinolytic therapy for breakthrough bleeding and as prophylaxis in patients with platelet transfusion refractoriness. 

### 2.2. Thrombopoeitin Receptor Agonists in Cancer and Thrombocytopenia

Thrombopoetin receptor agonists (TPO-RAs), such as eltrombopag and romiplostim, increase platelet production through interactions with the thrombopoietin receptor on megakaryocytes. Eltrombopag is a small molecule agonist, while romiplostim is a peptibody (i.e., fusion of a novel peptide and antibody) that can stimulate the TPO receptor. The binding of romiplostim to the distal domain of the thrombopoietin receptor or binding of eltrombopag to the transmembrane region of the receptor triggers a number of signal transduction pathways, including activation of the JAK-STAT signaling pathway, which induce proliferation and differentiation of megakaryocytes [70]. Eltrombopag and romipostim were both licensed in the United States for the treatment of immune thrombocytopenia in 2008. Eltrombopag is also licensed for the treatment of aplastic anemia and the treatment of thrombocytopenia in patients with hepatitis C receiving interferon-based therapy [71]. 

Recombinant IL-11 (oprelvekin) is the only approved treatment in the United States for CIT. However, its use is very limited because of side effects [72]. Clinical development of recombinant human thrombopoietins (rhTPO) and pegylated recombinant megakaryocyte growth and development factor (PEG-rhMGDF) have stopped due to the development of neutralizing antibodies to PEG-rhMGDF [73]. The rhTPO, TPIAO™, is widely used to treat CIT in China and is unavailable elsewhere [74].

TPO-RAs have been studied for two major implications in cancer related thrombocytopenia. In the field of hematological disorders, they were mainly studied for MDS and acute myeloid leukemia (AML), in order to treat severe thrombocytopenia and avoid platelet transfusions, as summarized in Table 2. In the field of solid tumors, they were used to prevent CIT and enable scheduled anti-cancer treatment. Prevention was either primary, before anti-cancer treatment, or secondary, after the development of thrombocytopenia. Selected studies on TPO-RAs in CIT are detailed in Table 3.

#### 2.2.1. Low-Intermediate Risk MDS

Giagouinidis et al. included 250 patients with low to intermediate (low-int) risk MDS to receive romiplostim or placebo (2:1) [75]. This study was terminated early because of an increase in peripheral blasts in the romiplostim group. Despite this initial signal, there was no increased risk of progression to AML in the romiplostim group [75], including in an analysis after five years follow-up [76]. Romiplostim increased platelet counts, and decreased platelet transfusions and overall bleeding, but did not affect clinically significant bleeding rates. Initial similar results were published for eltrombopag in low-int MDS [77]. That study reported improved quality of life in patients who received eltrombopag. The full study has not been published yet. Eltrombopag was also shown to increase white blood cell counts and hemoglobin levels in some patients in a small study of low-int risk MDS patients [78].

#### 2.2.2. High Risk MDS/AML

In a phase 1/2 study of advanced MDS or AML, eltrombopag was well tolerated in 64 patients, and no difference in the percentage of blasts was observed [79]. In a phase 3 trial of intermediate-high risk MDS treated with azacitidine, eltrombopag did not reduce the need for platelet transfusions. In fact, this study was terminated early due to inferiority of the eltrombopag/azacitidine arm (16% vs. 31%) and a trend towards increased progression to AML [80]. Furthermore, in a phase 2 placebo controlled trial of eltrombopag in patients with AML undergoing induction chemotherapy, eltrombopag did not decrease the time to platelet recovery, while more serious adverse events and numerically higher death rates were observed in the eltrombopag group [81].

#### 2.2.3. After Bone Marrow Transplantation

Persistent thrombocytopenia is a common complication after allogeneic hematopoietic cell transplantation. In a phase 1/2 single arm study, romiplostim given to patients after allogeneic stem cell transplantation who had persistent severe thrombocytopenia <20 × 10^9^/L (median of 84 days after transplantation), was effective in most patients. The median time to platelet counts >50 × 10^9^/L was 45 days [82]. Eltrombopag was also reported to achieve good platelet response in approximately 60% of patients in three small retrospective studies [83,84,85].

#### 2.2.4. High Grade Lymphoma

In a phase 1/2 open label in patients with Hodgkin or non-Hodgkin lymphoma, who experienced grade 3–4 thrombocytopenia (<50 × 10^9^/L), romiplostim given one day after chemotherapy did not have a beneficial effect on platelet nadir [86]. In contrast, in patients receiving the RHyper-CVAD/RArac-MTX protocol, romiplostim, given 5 days before and after chemotherapy, significantly (for a total of 2 doses) increased the platelet nadir and decreased the duration of thrombocytopenia [87]. 

#### 2.2.5. CIT in Solid Tumors

CIT in solid tumors is defined as platelet count below 100 × 10^9^/L with no other reason for thrombocytopenia. CIT may carry a risk of bleeding and may delay anti-cancer treatment and, therefore, it could potentially affect patients’ prognosis. A recent Cochrane review assessed the effects of TPO-RAs to treat and prevent CIT [88]. No certain conclusions could be made due to the weak available data. Selected studies for treatment of CIT are presented in Table 3. These were mostly retrospective studies that reported off-label use of romiplostim for this indication as well as several phase 2 studies. The main type of tumor was of gastrointestinal origin. Romiplostim rapidly increased platelet counts and could enable the scheduled anti-cancer treatments in most patients (Table 3). In the largest retrospective study to date, predictors of non-response to romiplostim included bone marrow tumor invasion, prior pelvic irradiation and exposure to temozolomide [89]. Nonetheless, in an open label phase II study of romiplostim in patients with glioblastoma receiving temozolomide, 60% of patients had good response and only 20% had no response [90]. The rate of thrombotic complications in patients who received romiplostim was reported between 5–15% (Table 3). Most of the events were VTE and only a small number of arterial events were reported. It is unclear whether TPO-RAs increase thrombosis in patients with cancer since no comparison group was included in most of the studies. A phase 3 study of avatrombopag vs. placebo in cancer patients who experienced grade 3–4 thrombocytopenia, was recently terminated due to futility, but is yet to be published. The press release reported that although avatrombopag increased platelet counts relative to placebo as expected, the study did not meet the composite primary endpoint of avoiding platelet transfusions, chemotherapy dose reductions by ≥15%, and chemotherapy dose delays by ≥4 days [91,92].

#### 2.2.6. Summary

TPO-RA studies in cancer are mainly retrospective or phase 2 trials. In these trials, both romiplostim and eltrombopag showed a potential benefit in patients experiencing severe thrombocytopenia related to low risk MDS and post allogeneic transplantation. In patients receiving chemotherapy for solid tumors TPO-RAs may improve platelet counts and the ability to prescribe scheduled anti-cancer treatments. The only two phase 3 trials of eltrombopag in patients with high risk MDS and avatrombopag in solid tumors did not meet the primary outcome. TPO-RAs may carry a risk in patients with advanced MDS in combination with azacitidine and in patients with AML undergoing induction chemotherapy. More phase 3 trials are indicated to investigate the role of TPO-RAs in cancer patients, some of which are planned or underway, as detailed in Table 4. 

## 3. Managing Antithrombotic Therapy in Thrombocytopenic Patients

Cancer is associated with an increased risk of both venous and arterial thrombosis [99,100,101]. Moreover, contemporary anticancer therapy and supportive care allow for the treatment of older patients with comorbid cardiovascular disease. This means that cancer patients, who are also at risk of thrombocytopenia, often have an indication for antithrombotic therapy (i.e., anticoagulation or antiplatelet therapy) before or after cancer diagnosis. Thrombocytopenic cancer patients remain at risk of venous and arterial thrombosis, since thrombocytopenia does not afford protection and is associated with adverse outcomes [102,103,104,105,106,107,108]. Multiple mechanisms, not dependent on the platelet compartment, contribute to cancer associated thrombosis, as recently reviewed [109]. These include tumor-driven increases in procoagulant activity and inhibition of fibrinolytic and natural anticoagulant pathways which lead to increased thrombin generation, as well as effects on leukocytes and endothelial cells. On the other hand, cancer patients are at increased risk of anticoagulation-associated bleeding [110,111], which is complicated by thrombocytopenia and other hemostatic defects [26,27,28]. Therefore, balancing the thrombotic and bleeding risk in thrombocytopenic risk remains a clinical challenge. Unfortunately, prospective data are scarce, meaning that management is currently informed mainly by expert opinion [112] and retrospective studies on VTE and ischemic heart disease [102,106,113,114,115,116], since clinical trials of anticoagulants in cancer-associated VTE exclude patients with thrombocytopenia (<50–100 × 10^9^/L) [117,118,119,120,121].

### 3.1. Management Concepts

We generally manage antithrombotic medication within the framework of international guidelines for treatment of VTE in thrombocytopenic cancer patients [122]. Importantly, these recommendations do not apply to other indications such as atrial fibrillation or antiplatelet medication, which generally lack specific guidelines. Therefore, we adjust management after considering context-specific evidence (see Section 3.2 and Section 3.3) and the risk-benefit ratio for the individual patient, bearing in mind the low level of evidence driving these recommendations.

#### 3.1.1. Risk Assessment

We always reevaluate the indication for antithrombotic therapy, and assess the associated thrombotic risk. We then estimate the anticipated duration of platelet counts below 50 × 10^9^/L, which may range from days to weeks in case of CIT or months to years for chronic disease-related thrombocytopenia, such as in MDS or graft versus host disease. Of note, the vast majority of evidence pertains to short-term thrombocytopenia. We also identify additional factors associated with higher bleeding risk in this setting, including a history of bleeding, hematological malignancy and increasing bilirubin, creatinine, and prothrombin time [113,114]. An important concept guiding management decisions is that these patients have a high short-term risk of clinically significant bleeding, especially with full anticoagulation [22,24,102,113,123,124,125]. Accordingly, the thrombotic risk should be sufficiently high to justify anticoagulation.

#### 3.1.2. Management Plan

Using the above information, we formulate a clear management plan, to be reassessed frequently, often on a daily basis. We first decide whether to continue or hold the antithrombotic medication. If continued, we consider changes in the dose and/or class of antithrombotic medication, and modifications in platelet transfusion thresholds. When anticoagulation is discontinued, mechanical measures to possibly mitigate thrombotic risk are considered on a case-by-case basis. These include inferior vena cava filter placement for acute lower extremity deep vein thrombosis (DVT) [122] or removal of the central venous catheter in case of catheter-related DVT [126]. Finally, once the platelet count is consistently above the threshold for full antithrombotic medication, we consider restarting full antithrombotic therapy, even between treatment cycles, if the indication remains [123].

### 3.2. Anticoagulation

Changes in anticoagulation management are generally recommended when platelets are <50 × 10^9^/L [112,122,127], since the bleeding risk appears to increase below this threshold [102,123]. The two main indications for therapeutic anticoagulation in this setting are VTE and atrial fibrillation. The evidence and guidelines relate almost exclusively to low-molecular weight heparin (LMWH). The lack of data on direct oral anticoagulants with platelets <50 × 10^9^/L, and increased bleeding risk even with prophylactic doses indicate that they should currently be avoided in this setting [119,121,122,128]. Retrospective cohort studies of VTE patients show varying bleeding and thrombotic rates, as summarized in a prior review [109].

The first month of anticoagulation for VTE is a high risk period for both recurrent bleeding and thrombosis [110], with higher rates of recurrent VTEs in populations enriched with acute VTE (i.e., within 30 days) [109]. Higher VTE burden (e.g., pulmonary embolism or proximal lower extremity DVT) is also considered to carry a higher thrombotic risk [122]. The CHA_2_DS_2_VASC score may be used to assess the thrombotic risk in patients with atrial fibrillation. Lower thrombotic risk scenarios where full-dose anticoagulation may not be justified include non-acute VTE (especially in autologous hematopoietic stem cell transplantation), catheter-related thrombosis and low risk atrial fibrillation [114,125,126,129]. Strategies for mitigating the high bleeding risk associated with continued anticoagulation include increased platelet transfusion threshold (e.g., 40–50 × 10^9^/L) and anticoagulation dose reductions, but evidence proving the safety and efficacy of both approaches is lacking [130].

Current guidelines use VTE acuity, risk of thrombus progression and platelet count to direct decisions regarding anticoagulation in thrombocytopenic cancer patients with VTE [122]. In case of acute VTE, high risk of thrombus progression and platelets <50 × 10^9^/L, increased platelet transfusion thresholds are recommended to enable full-dose anticoagulation. In patients with acute VTE and a lower risk of thrombus progression or those with non-acute VTE, LMWH dose reduction by 50% or prophylactic LMWH doses are recommended when platelets are 25–50 × 10^9^/L. Anticoagulation should generally be discontinued at platelet counts below 25 × 10^9^/L.

A recent study of 774 hypothetical case vignettes managed by 168 physicians suggested that the management process was compatible with these guidelines but that management varied according to physician characteristics and practice setting [131]. Of note, prior major bleeding and the type of hematological disease and treatment influenced management, and may be considered in the decision-making process, although not incorporated in the guidelines. Two recent retrospective analyses suggest that current management may achieve a reasonable balance between bleeding and thrombotic risk in VTE patients [116,129], but this remains to be confirmed prospectively by ongoing observational studies [132].

### 3.3. Antiplatelet Therapy

We generally discontinue aspirin used for primary prevention of arterial disease in patients with thrombocytopenia. The platelet threshold requiring changes in aspirin management appears lower than 50 × 10^9^/L, but the exact threshold is unknown [106,115]. There are sufficient data to suggest that aspirin use in acute myocardial infarction in thrombocytopenic patients (especially if platelets >30 × 10^9^/L) should be considered [106], but evidence on other indications is lacking.

Formal ischemic heart disease and stroke guidelines do not provide recommendations for management of thrombocytopenic cancer patients. In a consensus statement from the Society for Cardiovascular Angiography and Interventions (SCAI), aspirin was recommended when platelet counts were >10 × 10^9^/L, while dual antiplatelet therapy was reserved for platelets >30 × 10^9^/L [133]. A recent review, not specific to cancer, provided higher platelet thresholds (aspirin >50 × 10^9^/L; dual antiplatelet therapy >100 × 10^9^/L) [134].

A case vignette study assessing the decision-making process among 145 physicians across three countries, outlined the patient and physician factors influencing management. This study indicated that physicians considered ST elevation myocardial infarction to be a high-risk thrombotic scenario that warrants dual antiplatelet therapy despite thrombocytopenia [135]. Furthermore, platelet transfusion was used in 34% of cases continuing antiplatelet therapy to theoretically mitigate the risk of bleeding; however, there is no evidence to support this practice.

## 4. Summary

**Figure 2:**
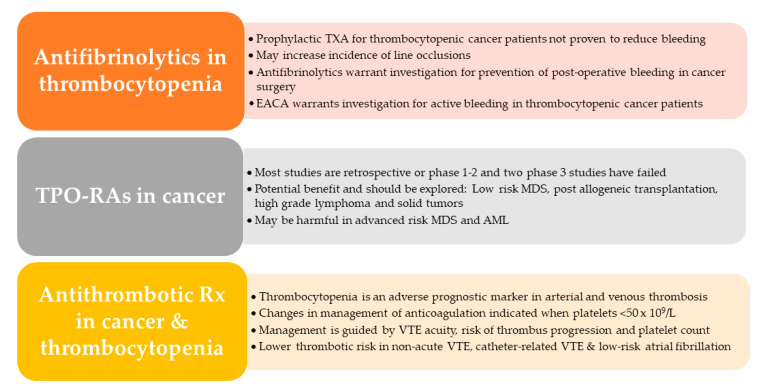
Take-home messages. *AML*, acute myeloid leukemia; *EACA,* epsilon aminocaproic acid; *MDS*, myelodysplastic syndrome; *TPO-RA*, thrombopoietin receptor agonists; *TXA*, tranexamic acid; *VTE*, venous thromboembolism.

The main take home messages regarding antifibrinolytics, TPO-RAs and antithrombotic medication in thrombocytopenic patients are shown in Figure 2. Platelet transfusion remains the cornerstone of managing thrombocytopenia in cancer, while we eagerly await the results of ongoing studies on antifibrinolytics and TPO-RAs.

## Figures and Tables

**Table 1:** Ongoing and planned clinical trials of antifibrinolytic agents in thrombocytopenic cancer patients ^1.^

Name, *Identifier*	Study Design (*Status*)	Interventions ^2^	Study Population ^3^	Primary Outcome	Time Frame	Planned Completion
**Antifibrinolytics in Thrombocytopenia**						
TRial to EvaluAte Tranexamic Acid Therapy in Thrombocytopenia (TREATT), *NCT03136445*	Interventional Randomized Phase 3 (*recruiting*)	***Arm A***: TXA 1 g q8hrs IV; ***Arm B***: TXA 1.5 g q8hrs PO.	Thrombocytopenic patients (platelet count of ≤10 × 10⁹/L for ≥ 5 days) with hematological malignancies (*n* = 616)	Death or bleeding (WHO grade ≥ 2)	30 days	March 2021
PRevention of BLeeding in hEmatological Malignancies with Antifibrinolytic (PROBLEMA), *NCT02074436*	Interventional Randomized Phase 2 (*recruiting*)	***Arm A***: EACA 1000 mg q12hrs; ***Arm B***: standard prophylactic platelet transfusion	Thrombocytopenic patients (platelet count < 20 × 10⁹/L) with hematological malignancies (*n* = 100)	Major bleeding episodes (WHO grades 3–4)	6 mo.	May 2021
Evolution of Thromboelastography During Tranexamic Acid Treatment (TTRAP-Bleeding), *NCT03801122*	Interventional Randomized Phase 2 (*recruiting*)	***Arm A***: TXA 3 g/day; ***Arm B***: TXA 1.5 g/day; ***Arm C***: No TXA	Thrombocytopenic patients (platelet count of ≤10 × 10⁹/L for ≥ 5 days) with hematological malignancies (*n* = 18)	Level of clot amplitude in thrombo-elastography	30 days	1 April 2022

^1^ Interventional phase 2 and 3 studies shown, as identified in https://www.clinicaltrials.gov/ (accessed on 31 January 2021). ^2^ The ratio between intervention arms is 1:1 or 1:1:1 unless otherwise specified. ^3^ All participants are aged 18 years or older, unless otherwise specified. *EACA,* epsilon aminocaproic acid; *IV*, intravenous; *PLT*, platelet count (×10^9^/L); *TXA*, tranexamic acid.

**Table 2:** Summary of studies on TPO-RAs in MDS and AML.

Design	Population	PLT, 10^9^/L	Intervention	TPO-RA Dose	Participants, *n*	Primary Outcome	Follow up	Results	Comments	Ref.
Phase 2 randomized	Low-int 1 MDS	<20	Romiplostim or placebo	250–1000 µg (750 µg start)	*n*(romi) = 167; *n*(placebo) = 83	CSBE	58 wks.	No difference in CSBE. Decreased overall bleeding. PLT increased. Transfusion reduced.	Early termination due to transient increase in peripheral blasts with romi. No increase in AML (18% vs. 20.5%)	[75]
					*n*(romi) = 139; *n*(placebo) = 83	OS and leukemic progression	5 years	No difference in OS or leukemic progression		[76]
Phase I of single blind randomized phase 2 trial	Low-Int 1 MDS	<30	Eltrombopag or placebo	50–300 mg	*n*(el-pag) = 59; *n*(placebo) = 39	Platelet response	11 wks. (median)	47% platelet response. Less bleeding	No difference in AML progression	[77]
Phase 2	Low risk MDS	<30	Eltrombopag	50–150 mg	25	Hematologic response	16 wks.	44% response	24% bi-lineage response	[78]
Phase 1/2	Advanced MDS or AML	<30	Eltrombopag or placebo	50–300 mg	*n*(el-pag) = 64; *n*(placebo) = 34	Safety & tolerability	6 mo. (optional 6 mo. extension)	Acceptable safety profile	No increase in marrow or peripheral blasts	[79]
Phase 3	Int-High risk MDS receiving azacitidine	<75	Eltrombopag or placebo	200–300 mg	*n*(el-pag) = 179; *n*(placebo) = 177	Platelet transfusion free	Cycles 1–4	More transfusion free with placebo (31%) than eltrombopag (16%). Worse PLT recovery	Terminated early due to futility and safety. Trend to AML progression.	[80]
Phase 2 double blind randomized	AML induction		Eltrombopag or placebo	200 mg	*n*(el-pag) = 74; *n*(placebo) = 74	Safety & tolerability	Until PLT > 200 or 42 days post-induction	More SAEs with eltrombopag	Numerically more deaths with eltrombopag. Same VTE rates.	[81]

*AML*, acute myeloid leukemia; *CSBE,* clinically significant bleeding events; *el-pag*, eltrombopag; *MDS*, myelodysplastic syndrome; *OS*, overall survival; *PLT*, platelet count; *romi*, romiplostim; *SAE*. Serious adverse events; *TPO-RA*, thrombopoietin receptor agonists; *VTE*, venous thromboembolism; *wk*, week.

**Table 3:** Selected studies on TPO-RAs for preventing or treating CIT in solid tumors.

Design	Cancer Type	PLT, 10^9^/L	Intervention	TPO-RA Dose	Participants, *n*	Primary Outcome	Follow up	Key Results	Comments	Ref
Retrospective cohort	Wide range, mostly GI	75 (median)	Romiplostim	3 µg/kg/wk (median starting)	22	ChemoRx dose delay and/or reduction	NR	Reduced dose delay (36% vs. 94%) and reduction	No thrombosis	[93]
Retrospective cohort	Wide range	<100 for ≥6 weeks (mean 58; range 3–97)	Romiplostim	2.9 µg/kg/wk (mean)	20	PLT > 100; ChemoRx delay	NR	95% PLT >100; 75% resumed ChemoRx without TCP	3 DVT patients (15%) could continue ChemoRx	[94]
Retrospective cohort	Wide range, mostly GI	<100	Romiplostim	2 µg/kg/wk (median starting)	37	PLT > 100; ChemoRx continued	18 wk (median)	95% PLT < 100; 92% continued ChemoRx	14% (n = 6) thrombosis. Mostly in pancreatic cancer (5/6)	[95]
Phase 2 randomized open label	Wide range, mostly GI	<100	Romiplostim or placebo	1 µg/kg/wk (starting)	*n*(romi) = 15; *n*(placebo) = 8; *n*(open-label-romi) = 37	PLT > 100 within 3 wks	6 wk	Overall response (n = 54) 85%	10% VTE	[96]
Phase 2	NSCLC with Gemcitabine and carboplatin/ cisplatin Rx	<50–100	Romiplostim or placebo	250–750 µg/wk	*n*(romi) = 50; *n*(placebo) = 12	Adverse events	≤5 cycles	Well tolerated. No effect on PLT or dose reduction	6% thrombosis	[97]
Retrospective cohort	Wide range	<100 for 3 weeks	Romiplostim	3 µg/kg/wk (median starting)	153	PLT response = Median PLT >75 or baseline +30	NR	71% response. 79% avoided dose delays or reduction	Less bleeding. 5.2% VTE (similar to historical controls)	[89]
Phase 2 open label single arm	Glioblastoma	<50	Romiplostim	750 µg/wk (starting; dose adjusted)	20	Proportion completing 6 cycles	6 cycles	60% completed 6 cycles	5% lower limb ischemia; 5% VTE	[90]
Phase 2	Advanced solid tumors, with Gemcitabine ± Carboplatin Rx	<150	Eltrombopag or placebo	100 mg/day	*n*(el-pag) = 53; *n*(placebo) = 23	PLT pre + post ChemoRx	6 cycles	Less time to PLT recovery; Fewer dose delays or reduction	Reduced rates of anemia and leukopenia	[98]
Phase 3	Ovarian, small cell lung cancer, NSCLC, bladder cancer	<50 in a previous treatment cycle	Avatrombopag or placebo	60 mg/day (5 days pre and post Rx)	122 (ava-pag:placebo, 2:1)	PLT transfusion or dose delays or reduction		No difference between ava-pag and placebo	Higher PLT in ava-pag group	[91,92]

*ava-pag*; avatrombopag; *ChemoRx*, chemotherapy; *CIT*, chemotherapy-induced thrombocytopenia; *DVT*, deep vein thrombosis; *el-pag*; eltrombopag; *GI*, gastrointestinal; *NR*, not reported; *NSCLC*, non-small cell lung cancer; *PLT*, platelet count; *romi*, romiplostim; *Rx*, treatment; *TCP*, thrombocytopenia; *TPO-RA*, thrombopoietin receptor agonists; *VTE*, venous thromboembolism; *wk*, week.

**Table 4:** Ongoing and planned clinical trials of TPO-RAs in thrombocytopenia and cancer ^1^.

Name, *Identifier*	Study Design (*Status*)	Interventions ^2^	Study Population ^3^	Primary Outcome	Time Frame	Planned Completion
TPO-RAs in Hematological Malignancies						
Eltrombopag for the Treatment of Thrombocytopenia Due to Low- and Intermediate Risk Myelodysplastic Syndromes, *NCT02912208*	Interventional Randomized Phase 2 (*recruiting*)	***>Arm A***: eltrombopag 50–300 mg/day; ***Arm B***: placebo	Stable low or int 1 MDS with PLT < 30, ineligible to receive other treatment options (*n* = 174)	Platelet response rate (complete or any)	6 mo.	August 2019
Phase II Study of Lenalidomide and Eltrombopag in Patients with Symptomatic Anemia in Low or Intermediate I Myelodysplastic Syndrome (MDS), *NCT01772420*	Interventional non-randomized Phase 2 with parallel assignment (*recruiting*)	***Arm A*** if PLT > 50: lenalidomide and eltrombopag; ***Arm B*** if PLT < 50: eltrombopag until ***PLT*** > 50 for 2 wks. Then, Arm A.	Low-int 1 MDS or chronic myelomonocytic leukemia (*n* = 60)	Hematologic improvement as defined by the IWG 2006 criteria	8 weeks	January 2020
Eltrombopag in Myelodysplastic Syndrome (MDS) Patients with Thrombocytopenia, *NCT01286038*	Interventional single arm Phase 1–2 (*active, not* *recruiting*)	Eltrombopag	MDS after hypomethylating agent failure and PLT < 50 (*n* = 37)	Maximum tolerated dose	24 mo.	September 2021
Validation of a predictive model of response to romiplostim in patients with IPSS low or intermediate-1 risk MDS and thrombocytopenia (EUROPE-trial),	Interventional non-randomized Phase 2 with parallel assignment (*recruiting*)	Romiplostim stratified using TPO-based model to ***Arm A*** (score +3), ***Arm B*** (−1 or −2), ***Arm C*** (−6)	Low or int 1 MDS with PLT < 30 or PLT < 50 and bleeding (*n* = 84)	Hematologic improvement of platelets (HI-P) after 4 months on therapy	12 mo.	December 2021
Eltrombopag Olamine in Treating Thrombocytopenia in Patients with Chronic Myeloid Leukemia or Myelofibrosis Receiving Tyrosine Kinase Therapy, *NCT01428635*	Interventional single arm Phase 2 (*active, not recruiting*)	Eltrombopag	CML or MF patients with platelets <50 × 10^9^/L (CML) or <100 × 10^9^/L (MF) after 3 mo. of TKIs (*n* = 39)	Complete platelet response	30 days after last dose of eltrombopag	31 January 2022
Using Romiplostim to Treat Low Platelet Counts Following Chemotherapy and Autologous Hematopoietic Cell Transplantation in People with Blood Cancer, *NCT04478123*	Interventional single arm Phase 2 (*recruiting*)	Romiplostim 3 µg/kg on day 1+ after transplant (start dose)	Patients with multiple myeloma or lymphoma undergoing high dose chemotherapy with autologous stem cell transplant (*n* = 60)	No. of days post-transplant requiring platelet transfusions or grade 4 thrombocytopenia	42 days	July 2022
Using Romiplostim to Treat Low Platelet Counts during Chemotherapy in People with Lymphoma, *NCT04673266*	Interventional single arm Phase 2 (*recruiting*)	3 µg/kg/wk. on day 1 of chemotherapy cycle, titrated weekly	Lymphoma patients receiving chemotherapy, with grade 4 thrombocytopenia during previous cycle or PLT < 50 on day 1 of current cycle (*n* = 20)	PLT indication for dose delay (see definition)	1 year	December 2022
Study Impact on Outcome of Eltrombopag in Elderly Patients with Acute Myeloid Leukemia Receiving Induction Chemotherapy (EPAG2015), *NCT03603795*	Interventional Randomized Phase 2 (*recruiting*)	***Arm A***: eltrombopag 200 mg/day; ***Arm B***: placebo	Patients aged ≥ 60yrs with de novo AML eligible for intensive induction chemotherapy (*n* = 110)	Overall survival rate	12 mo.	September 2024
**TPO-RAs for CIT in solid tumors**						
A Study of Romiplostim to Prevent Low Platelet Counts in Children and Young Adults Receiving Chemotherapy for Solid Tumors, *NCT04671901*	Interventional single arm Phase 2 (*recruiting*)	3 µg/kg/wk. from cycle 4, titrated weekly	Patients aged 1–21 years with a primary solid tumor undergoing N8/EFT chemotherapy treatment (*n* = 30)	No. of platelet transfusions	6 mo.	10 December 2022
Study of Romiplostim for Chemotherapy-induced Thrombocytopenia in Adult Subjects with Gastrointestinal, Pancreatic, or Colorectal Cancer (RECITE), *NCT03362177*	Randomized double-blind placebo controlled Phase 3 (recruiting)	***Arm A***: romiplostim 3 µg/kg/wk., titrated weekly; ***Arm B***: placebo (2:1 ratio)	Patients receiving oxaliplatin-based chemotherapy for gastrointestinal/ colorectal/ pancreatic cancer, with PLT < 75 at or after scheduled start of next cycle (*n* = 162)	Thrombocytopenia-induced chemotherapy dose modification during the second or third on study chemotherapy cycles	48 days	1 June 2023
Study of Romiplostim for Chemotherapy-induced Thrombocytopenia in Adult Subjects with Non-small Cell Lung Cancer (NSCLC), Ovarian Cancer, or Breast Cancer, *NCT03937154*	Randomized double-blind placebo controlled Phase 3 (*recruiting*)	***Arm A***: romiplostim; ***Arm B***: placebo (2:1 ratio)	Patients receiving carboplatin-based chemotherapy for locally advanced or metastatic non-small cell lung cancer, ovarian cancer, or breast cancer, with PLT < 75 at or after scheduled start of next cycle (*n* = 162)	Chemotherapy dose delay or reduction	48 days	28 June 2023
Avatrombopag on the Treatment of Thrombocytopenia Induced by Chemotherapy of Malignant Tumors, *NCT04609891*	Interventional single arm Phase 2 (*recruiting*)	Avatrombopag 40 mg or 60 mg, depending on PLT. Duration differs between prevention and treatment	Patients with solid tumors receiving chemotherapy, and 10 < PLT < 75 after the last cycle (*n* = 80)	Chemotherapy dose delay or reduction, or platelet transfusion	2 mo.	31 August 2021

^1^ Interventional phase 2 and 3 studies shown, as identified in https://www.clinicaltrials.gov/ (accessed on 31 January 2021). ^2^ The ratio between intervention arms is 1:1 or 1:1:1 unless otherwise specified. ^3^ All participants are aged 18 years or older, unless otherwise specified. *AML,* acute myeloid leukemia; *CIT,* chemotherapy induced thrombocytopenia; *CML*, chronic myeloid leukemia; *MDS*, myelodysplastic syndrome; *MF*, myelofibrosis; *mo.,* months; *PLT*, platelet count (x10^9^/L); *TKI*, tyrosine kinase inhibitor; *TPO-RA*, thrombopoietin receptor agonist.
